# Analysis of Low-Velocity Impact Resistance of Carbon Fiber Reinforced Polymer Composites Based on the Content of Incorporated Graphite Fluoride

**DOI:** 10.3390/ma13010187

**Published:** 2020-01-02

**Authors:** Jing Leng, Tianzi Guo, Meng Yang, Zeshi Guo, Zhengqin Fang, Zhipeng Liu, Dandan Li, Dazhi Sun

**Affiliations:** Department of Materials Science and Engineering, Southern University of Science and Technology, Shenzhen 518055, China; lengj@sustech.edu.cn (J.L.); guotz@mail.sustech.edu.cn (T.G.); yangm@mail.sustech.edu.cn (M.Y.); guozs@mail.sustech.edu.cn (Z.G.); fangzq@mail.sustech.edu.cn (Z.F.); liuzp@mail.sustech.edu.cn (Z.L.); lidd@sustech.edu.cn (D.L.)

**Keywords:** CFRP composites, impact resistance, graphite fluoride

## Abstract

As a graphite derivative, graphite fluoride (GrF) has a remarkable fracture toughness improvement effect on epoxy materials. The fracture toughness variation of the epoxy could exert an influence on the low velocity impact resistance of the corresponding carbon fiber reinforced polymer (CFRP) composite. Therefore, the dependence of the low velocity impact resistance of the incorporated CFRP on the GrF content is worth analyzing. Here, different contents of GrF were applied to incorporate CFRP laminates and planned to find the optimal GrF content, in turn leading to the best impact resistance. Using a drop-weight impact test, the load vs. time curves and load vs. displacement curves were obtained. The incipient damage loads and maximum loads of various GrF contents of the samples were compared carefully. The absorbed energies during the impact process were calculated. The trend of absorbed energy decreased up to the 1 wt% sample, then increased significantly with the rise of GrF content. This deflection behavior can be explained by the combination of crack pinning, crack deflection and crack propagation, due to the rise in GrF content. Through the ultrasonic C-scan evaluation, the delamination areas of different GrF content of samples were measured. The trend of delamination area variation was accordant with the trend of absorbed energy variation. This presents a demonstration of the correlation between the absorbed energy and the damage level. The SEM images of the fracture surfaces were analyzed for the deflection behavior of the fracture toughness with various GrF contents. The plot of residual compression strength versus GrF content further indicated the 1 wt% was the optimal content at which the incorporated GrF endowed the most impact-resistant property to the CFRP laminates.

## 1. Introduction

As one of the most extensively used fiber-reinforced composite materials, carbon fiber reinforced polymer (CFRP) shows tremendous potential to replace traditional metal materials in aviation, and naval and automotive fields, due to its lighter weight, higher specific strength and better corrosion resistance. For instance, in the aviation industry, CFRPs are capable of constituting almost 50% of the Boeing 787 airframe, with a 20% weight saving [1]. In the automotive field, the annual consumption of carbon fiber is estimated to be around 23,000 tonnes by 2020 [2]. In the wind energy blade field, carbon fiber demand had grown to 25,000 tonnes/year by 2018 [3]. As the novel fiber-reinforced composite technology advances, CFRP materials will be used more widespread and in-depth.

Since the carbon fiber possesses a higher specific elastic modulus and higher specific strength than metal materials [4], the derived CFRPs have a comparable mechanical strength to metal materials of that magnitude [5]. However, for the reason of the structure of CFRPs—the brittle epoxy matrix accounts for a considerable proportion of the CFRP material, the overall impact resistance is attenuated significantly in contrast to the pure carbon fiber. Unlike metal materials, the impact on CFRPs not only causes barely visible impact damage (BVID), but also produces internal damage under the surface [6]—which is harder to inspect. The latter will degrade the residual mechanical property of materials and lead to a sudden failure without warning when the materials are in service. Based on the different velocity levels and stress wave responses, the impact events can be categorized into low velocity impact, intermediate velocity impact and high velocity impact [7,8]. When the impact velocity is as high as a critical velocity, the stress wave propagation effect cannot be neglected. Davies et al. [9] proposed a simple model to determine the critical velocity, where if the impact velocity reaches it, the stress wave effect will predominate in the stress distribution. According to this model, the low velocity impact is defined as the impact event whose impact velocity is not more than 10 m/s [10]. In contrast to the two other impacts, the low velocity impact allows the target enough time to respond and no stress wave propagation effect needs to consider. The target will fail as a result of flexural response to the impact damage [11]. The failure is a combination of manifold damage mechanisms such as matrix cracking, delamination and fiber breakage [12,13]. The mechanical properties of fiber and matrix, especially the failure strain and interface properties, are of importance in target impact damage resistance and damage tolerance [14]. The nanofiller modified polymer nanocomposites can improve material impact properties effectively through suppressing subcritical cracks in the matrix [15]. Reis et al. found nanoclay Cloisite 30B-modified Kevlar fiber/epoxy composite had a higher maximum load increased by 16% and lower displacement [16]. Dispersed nanoclay filler can give rise to significant improvements in impact fracture toughness, such as Nanomer I30P-modified CFRPs [17]. 

Graphite fluoride (GrF), as one of the graphite derivatives, has a great possibility to become a novel widespread functional nanomaterial. Theoretically, GrF possesses the lowest surface energy [18,19,20]—relying on this feature, it can be incorporated in polyimide matrix to enhance the wear life of polymer tools [21]. In a reinforcement aspect, GrF displays a favorable strengthening effect in PA6/GrF composites [22], and remarkable fracture toughness improvement in GrF/epoxy composites [23]. To the best of our knowledge, there is a correlation between the high fracture toughness and superior impact damage resistance [3,24]. So GrF should be a good candidate to strengthen CFRPs impact damage resistance and tolerance.

In this work, different contents of GrF-modified CFRP composite laminates were fabricated from 0 wt% to 4 wt%. To more carefully confirm which content allowed for the best impact resistance improvement effect, we adopted only one impact energy level to test the composite laminates, so as to focus on the analysis of impact process data and post-impacted results in more detail. The impact energy level was selected beforehand, which was selected to be sufficient to create damage, in order to produce complementary data and, at the same time, to avoid too severe damage to make a comparison between the post-impacted results difficult. So, we applied 18 J impact energy to test the GrF-modified CFRP composite laminates and collected the impact load data and displacement data along with time variables, then calculated the absorbed energy and measured the delamination area by scanning acoustic microscopy (SAM). Furthermore, the compression after impact test was carried out to evaluate the damage tolerance of the composite laminates, to establish the correlation between the residual compression strength and the variation of GrF content.

## 2. Experimental

### 2.1. Materials and Fabrication of Composite Laminates

#### 2.1.1. Materials

The composite laminates were manufactured from plain-woven carbon fiber and graphite fluoride filled epoxy resin. The epoxy resin system was supplied by Wells Advanced Materials Corp. An epoxy matrix based on resin (LT-5028A) and hardener (LT-5028B) were used at a ratio of 100:30 by weight. The plain-woven carbon fiber (T300, 3 K, 200 g/m^2^) was supplied by Toray Composite Materials America, Inc. as the main reinforcement for composite laminates. The GrF (SV-B) powders (2.5 g/cm^3^) with a particle diameter of about 5 µm and C:F = ~1:1 were supplied by Shenyang Xi Fu technology Co., Ltd. (Shenyang, China).

#### 2.1.2. Fabrication of Composite Laminates

The GrF was added to epoxy hardener with different weight content (from 0 wt% to 4 wt% of the final resin-hardener mixture) and stirred for 30 min to obtain a uniformly distributed GrF/hardener precursor. The precursor was poured to a corresponding amount of epoxy resin and stirred quickly to get a homogeneous mixture. Then the mixture was degassed in vacuum oven to remove all dissolved air bubbles. Ten plies of identical plain-woven carbon fiber fabrics were prepared by hand lay-up on a glass mould plate beforehand. The fabrication of composite laminates was performed via a typical vacuum-assisted resin transfer molding process (VARTM). A low pressure of 0.26 MPa was applied to maintain a constant fiber volume fraction and a uniform laminate thickness of 2.5 mm. The curing process was performed by manufacturer’s recommended cure cycle, 4 h dwell at 50 °C and 6 h dwell at 70 °C.

### 2.2. Methods and Characterization Techniques

#### 2.2.1. Drop-Weight Impact Test

**Figure 1:**
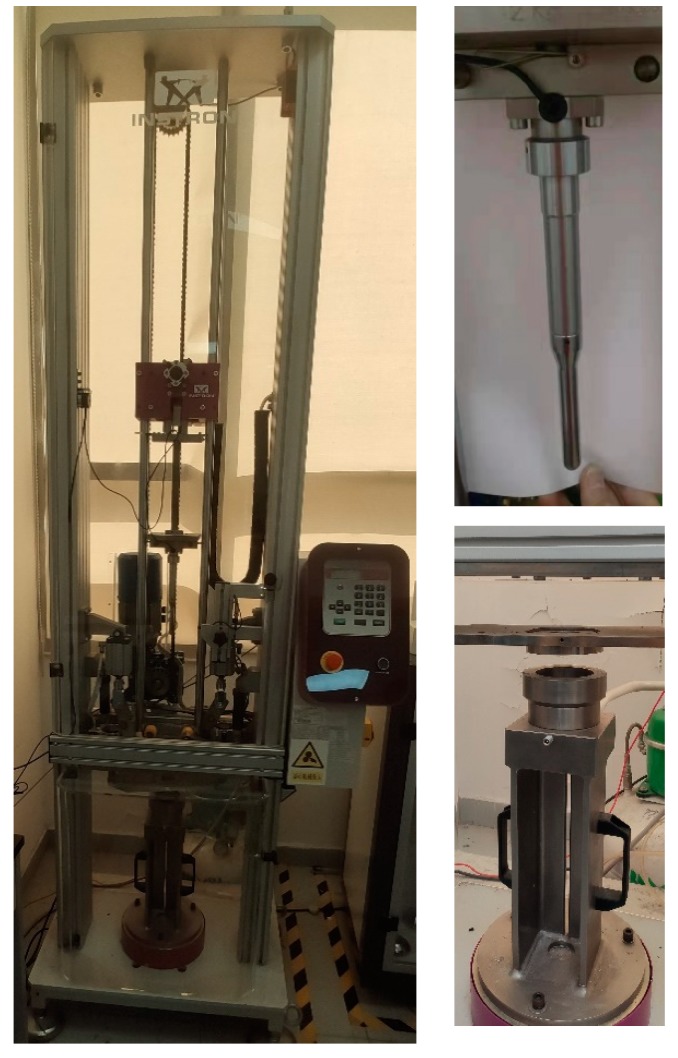
The impact test setup.

Impact tests were carried out on 100 mm × 100 mm square samples, at room temperature on an Instron CEAST 9340 Drop Weight Impact Tester in Shenzhen Academy of Aerospace Technology (Shenzhen, China), as shown in Figure 1. The impact force of the impactor with a hemispherical nose of 20 mm diameter and weight of 2.9 kg was measured during the impact process. The composite laminates were fixed firmly between two circular rings with a 75 mm diameter test window. The initial impact velocity of the impactor was observed by an optical cell. The incident impact energy was determined by the height between the impactor and composite laminate samples. The energy was calculated with the equation *E* = *mgh*, where *E* is the impact energy, *m* is the mass of the impactor, *g* is the acceleration due to gravity, and *h* is the drop height. The system Instron CEAST DAS 64K was employed to acquire data with frequencies up to 4 MHz. The pneumatic rebound brake avoided multiple impacts on the laminate samples.

#### 2.2.2. C-Scan Evaluation of Impact Damage

The interface delamination caused by impact damage was characterized using a C-mode scanning acoustic microscope (C-SAM, Sonoscan GEN6) by Integrated Service Technology (Shenzhen) Co. Ltd. The scanning was done in pulse–echo immersion mode using a transducer with 75 MHz probe. The gate was set on the back-surface echo. By monitoring the amplitude of the ultrasonic echo signals at specific depth of the laminates, the ply-by-ply C-scan images were obtained along y-axis. Both the front and back sides of the damage areas of the tested laminates were evaluated with Sonolytics^™^ software.

#### 2.2.3. Compression after Impact (CAI) Tests

The compression after impact (CAI) tests were operated on a SANS CMT7504 universal testing machine in Shenzhen Academy of Aerospace Technology. The ASTM D7137/D7137M-17 test method [25] was employed to evaluate the damage tolerance of CFRPs, that is the residual compressive strengths suffered the specified impact energy level. The CAI test uses an anti-buckling system, and the top and bottom edges of the samples were aligned parallel with each other and perpendicular to the loading direction. The cross-head speed was set to 1.0 mm/min at room temperature. The residual compressive strength and the original compressive strength were both measured using this CAI test.

## 3. Results and Discussion

### 3.1. Characterization of Impact Loads and Energies

**Figure 2:**
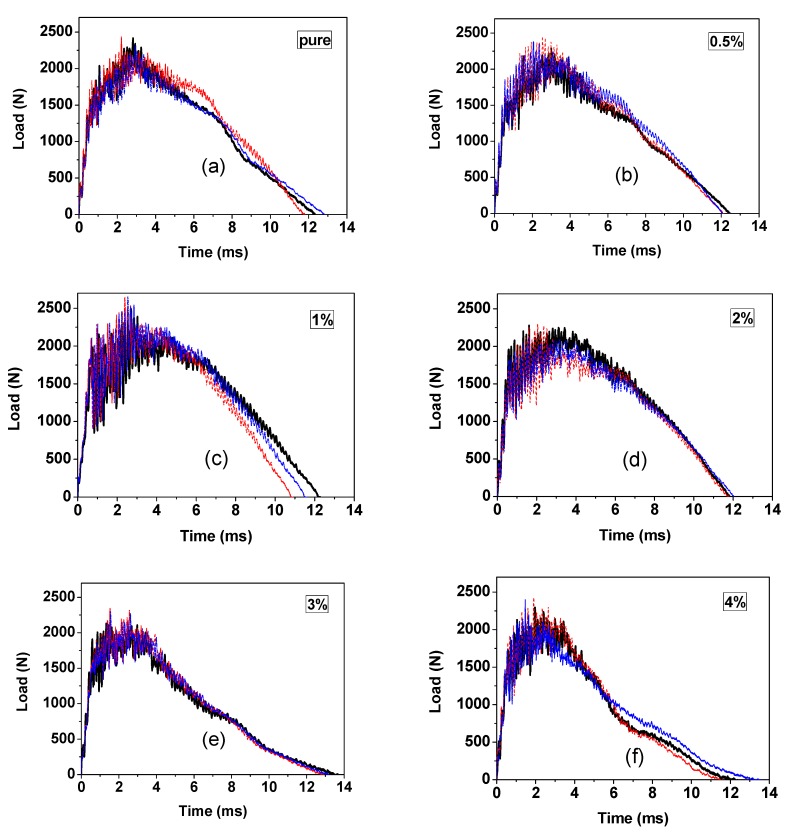
Load vs. time curves of the graphite fluoride (GrF)-modified carbon fiber reinforced polymer (CFRP) composites with (**a**) 0 wt%, (**b**) 0.5 wt%, (**c**) 1 wt%, (**d**) 2 wt%, (**e**) 3 wt%, (**f**) 4 wt% content at 18 J impact energy. For accuracy, every content of sample was measured three times; all results were plotted by different colors.

**Figure 3:**
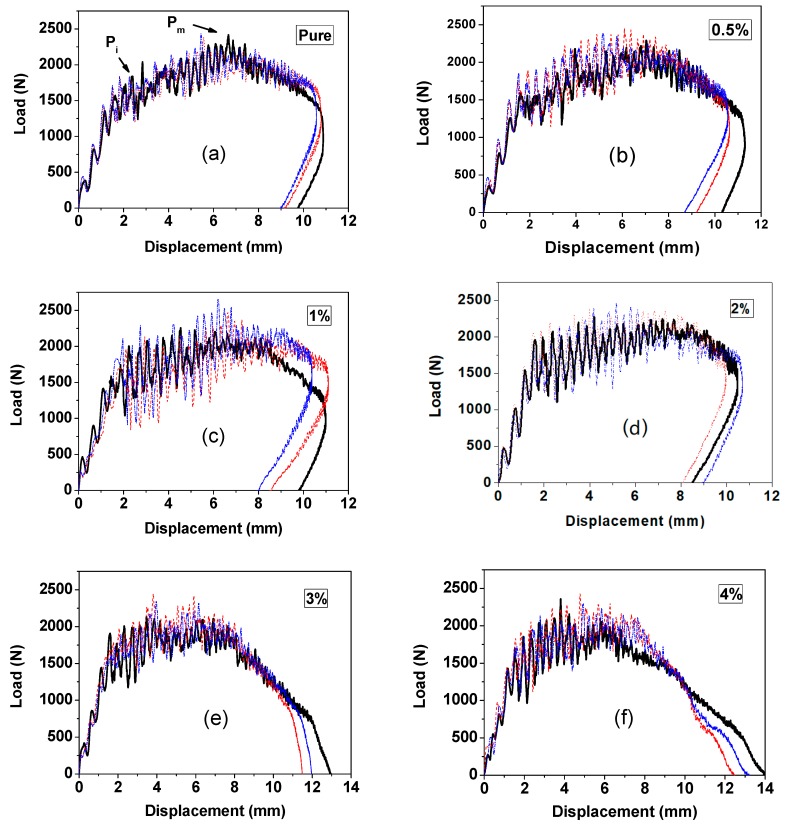
Load vs. displacement curves of the GrF-modified CFRP composites with (**a**) 0 wt%, (**b**) 0.5 wt%, (**c**) 1 wt%, (**d**) 2 wt%, (**e**) 3 wt%, (**f**) 4 wt% content at 18 J impact energy. For accuracy, every content of sample was measured three times; all results were plotted by different colors.

The characterization of the impact resistance of GrF-modified CFRP composite material involves the measurements of incipient damage load, *P_i_*, maximum load, *P_m_* (as shown in Figure 3), and load–time curves, etc. The incipient damage load can be identified by the first sudden load drop [26], in other literatures, it was also called Hertzian failure [27,28,29] or the delamination threshold load [30], which corresponds to the initial damage of the matrix cracking, fiber breakage and delaminations. The value of the incipient damage load can be used to determine the impact resistance of CFRP composite materials, because the incipient damage load (*P_i_*) is a constant value in relation to the specific material. Davies et al. [31] proposed a quantitative equation to obtain the value of incipient damage load, *P_i_* = 8*π*^2^*EhG_IIc_*/9(1 − *ν*^2^), where *E* is the equivalent in-plane modulus and *ν* is the Poisson ratio, *h* and *G_IIc_* are the laminate thickness and the critical strain energy release rate, respectively. Beyond the incipient damage load, the residual compression strength will decrease dramatically due to the occurrence of delaminations. The maximum load (*P_m_*) represents the highest load value the CFRP composite laminates can withstand during the impact process. The maximum load is determined by the impact energy level and the material strength, since for a specific structure the load history only depends on the impact energy level rather than the mass or the velocity [32]. In our case, the sole 18 J impact energy is applied, which is sufficient to cause considerable damage. For the damaged case, the load rises to the peak then falls to zero with a longer time compared to the former rising phase. The non-damaged cases normally show a sinusoidal, simple harmonic motion load history with symmetrical equal time. The slope of the load-time curves represents the contact stiffness of the CFRP composites—the steeper slope indicates a higher contact stiffness [33]. As shown in Figure 2, before the maximum load, the slopes of all composite samples keep the same. After the maximum load, with the damage already formed, the slopes change depending on the different GrF contents. It depicts the 1 wt% and 2 wt% GrF-modified CFRP composites as having the steepest decay slope, then the pure and 0.5 wt% composite samples having a moderate slope, and the 3 wt% and 4 wt% samples as having the most gradual slope. This suggests that the 1 wt% or the 2 wt% content sample should have the highest contact stiffness.

**Figure 4:**
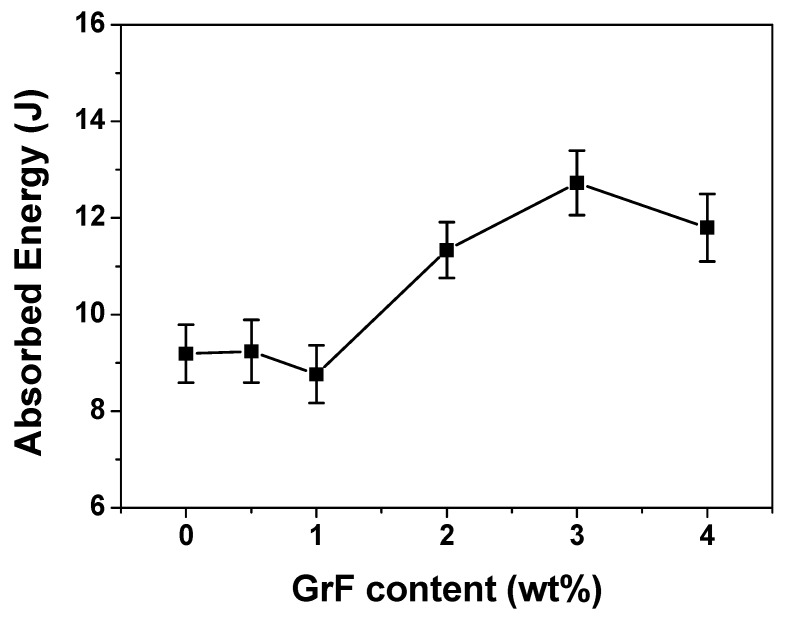
The average absorbed energy vs. GrF content curve of the CFRP composites, when the impact energy is 18 J.

Figure 3 gives the load-displacement curves for all contents of GrF-modified CFRP. The incipient damage load (*P_i_*) can be found in the plots. The displacement represents the distance of the impactor penetrating through the laminate samples from the initial contact position. The integral area under the load-displacement curve can be considered as the work the composite laminates applied onto the impactor. In general, the energy absorbed by the samples is designated as the difference between the energy at the end of the impact process and the energy at the maximum load point [34]. Because the CFRP composites are rigid, the energy before reaching the maximum load is considered to be absorbed by the elastic deformation, only the energy after the maximum load is devoted to producing damage. The absorbed energy can be used to evaluate the damage level of the post-impacted laminates. Figure 4 gives the curves absorbed energy versus GrF content. The 1 wt% sample displays the lowest absorbed energy, which should correspond to the lowest damage level. As shown in Figure 4, the absorbed energy value decreases at 1 wt%, then increases depending on the raising GrF content, at 4 wt% the value turns down slightly. This behavior should be related to the fracture toughness variation with the increasing incorporated filler loading [35,36]. When the filler loading is small, the microvoid formed around the filler can generate crack pinning and crack deflection effects to toughen the overall material. On the other hand, with the raising concentration of the microvoid, as the microcrack zones become closer and closer to each other, a major crack propagation could be promoted accordingly. When the filler content is at a very low level, the toughening effect resulting from crack pinning and crack deflection predominates, whereas above a critical content of filler, the major crack propagation overwhelms the toughening effect, then the fracture toughness of the matrix material is lowered. The fracture toughness strengthening was considered as one of the most important factors to improve the low velocity impact resistance of epoxy-based composite materials [37,38,39]. As shown in Figure 3, the 3 wt% and 4 wt% samples have the biggest displacement at the end of impact process, and no rebound compared to the content of the other samples, which indicates that under the same impact level, the perforation of 3 wt% and 4 wt% samples reach the largest depth, and the material strength was reduced to a level even too insufficient to form rebound.

**Figure 5:**
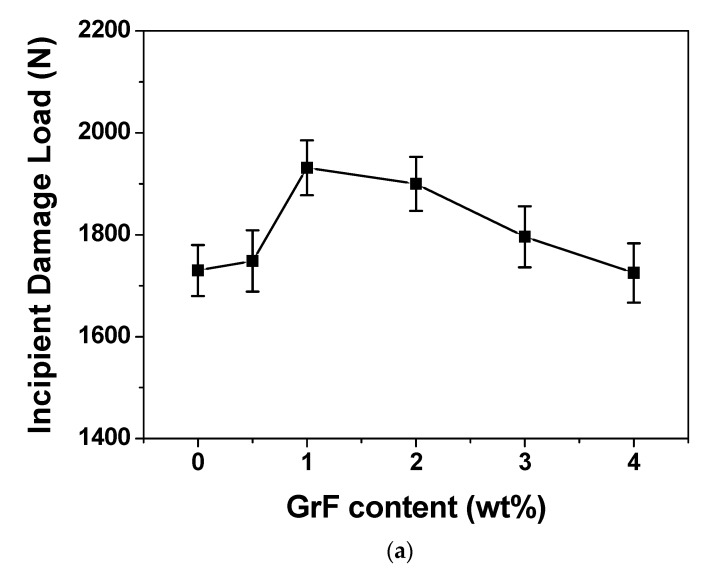

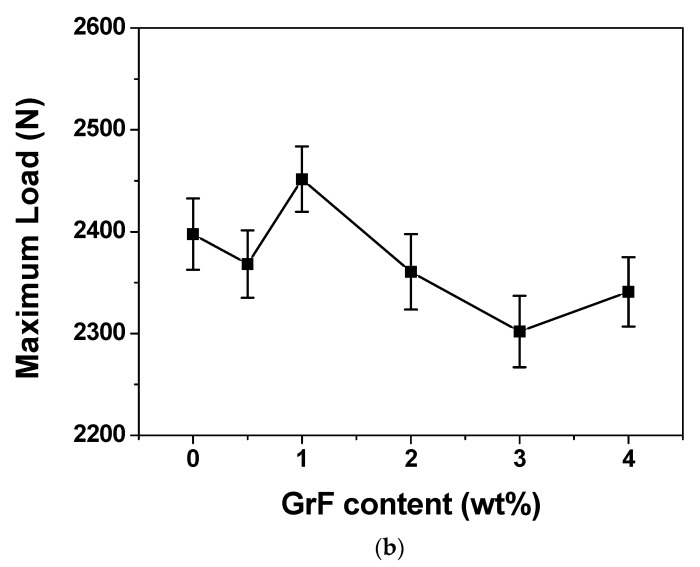
(**a**) The average incipient damage load and (**b**) average maximum load vs. GrF content curves of CFRP composites.

Figure 5a,b give the incipient damage load (*P_i_*) and the maximum load (*P_m_*) of all contents of the composite laminate samples. The 1 wt% sample owns the highest *P_i_* and highest *P_m_*, since the *P_i_* is the load threshold of the initial damage and *P_m_* is the load threshold the material can withstand during impact process, higher values for *P_i_* and *P_m_* represent higher impact resistance.

The curves of *P_i_* vs. GrF content and *P_m_* vs. GrF content both show a deflection point. Beyond 1 wt%, the *P_i_* and *P_m_* both descend, which indicates that the impact resistance also descends. This behavior is coincident with the absorbed energy variation with GrF content, as shown in Figure 4. The mechanism behind this behavior involves crack pinning, crack deflection and crack propagation caused by the concentration variation of microcrack zones. It is obvious around 1 wt% that the impact resistance improvement effect of GrF to CFRP composite laminates reaches a maximum level, as when exceeding 1 wt% the crack propagation gains advantage, which leads to the fracture toughness lowering and, in turn, the impact resistance worsening.

### 3.2. Characterization of Impact Damage Areas

The damage patterns were characterized by ultrasonic C-scan technique. The damage pattern could contain all possible damage modes including delamination, buckling, matrix cracking, fiber fracture and perforation [24,33,40]. However, in our case, all samples were tested at the same impact energy level, and all composite laminates were perforated during the impact process, so the damage levels were very similar. According to the size of the perforation holes on the front side of post-impacted laminates, there was no observed difference. The fiber fracture damage levels are largely dependent on the impact energy levels for their large damage threshold energy, since the impact energy level is identical, the fiber fracture conditions are not much different. As mentioned previously, the major difference of the CFRP composite laminates is the GrF content variation, which gives rise to the matrix fracture toughness fluctuation, and influences the impact resistance of the laminate samples. Joshi and Sun et al. established the correlation between the delamination area and the matrix crack propagation [41]: on the woven carbon fiber fabric composite, when the cracks due to the impact emerge from the upper layer, they branch into transverse and longitudinal directions—the cracks in the transverse direction could restrict the upper interface delamination and, meanwhile, fade away from the impact region, the cracks in the longitudinal direction initiate the lower interface delamination and with the delamination propagation the upper layer transverse cracks spread downward then interact with the expanding lower interface delamination, with the delamination area becoming larger around the more intense cracks. On the basis of the above mechanism, a qualitative correlation that the post-impacted delamination area is proportional to the crack propagation level can be determined.

**Figure 6:**
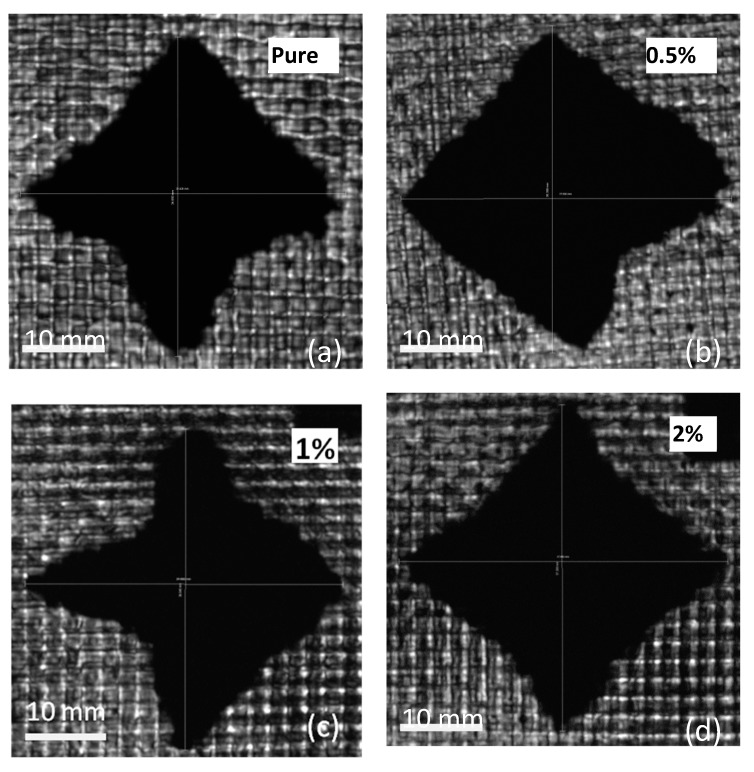

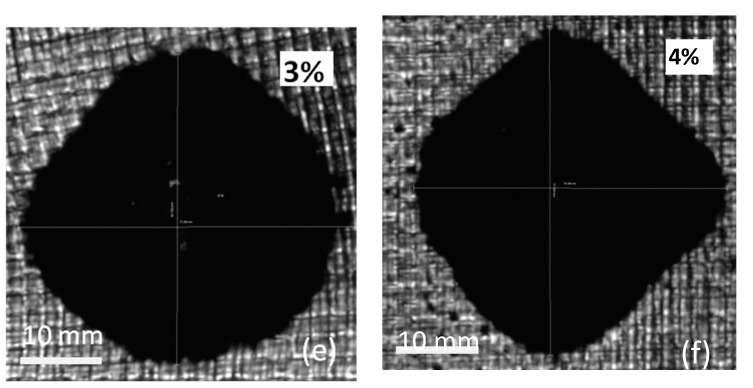
The C-scan images of back side of the post-impacted laminate samples.

**Figure 7:**
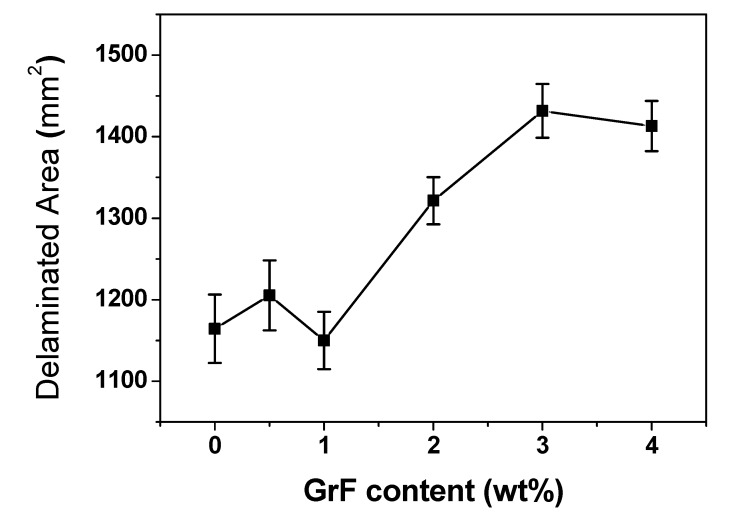
The average delamination area of post-impacted CFRP laminates with various GrF contents.

Figure 6 depicts the C-scan images of the back side of the post-impacted CFRP, the images display specific depth profiles of the damage through reflected ultrasonic pulses. The delamination patterns of laminates are different with various GrF contents. The pure, 0.5 wt% and 2 wt% samples all display a half-rhombus and half-cruciform shape. The 1 wt% sample has a more approximating cruciform shape in contrast to the other content samples, while the 3 wt% and 4 wt% samples have a rectangle-like shape. The shapes of delamination pattern imply that the crack propagation intensity is different along the woven fabric orientation. As previously mentioned, the cracks spread along the fabric orientations and as they initiate and interact with the interface delaminations, the lower matrix fracture toughness produces a larger delamination area, thus the 3 wt% and 4 wt% samples show a plumper delamination shape with a similar length-to-width ratio, while the 1 wt% sample exhibits the slimmest shape for its highest matrix fracture toughness. The delamination areas were measured in Figure 7 with various GrF contents of the composite samples. The trend of delamination area variation coincides with absorbed energy variation as shown in Figure 4—they almost have the same tendency at each corresponding GrF content. This coincidence renders a clear demonstration of the correlation between the absorbed energy and the damage level. Based on the coincident tendency, a quantitative dependence of delamination area on the absorbed energy could be expected.

**Figure 8:**
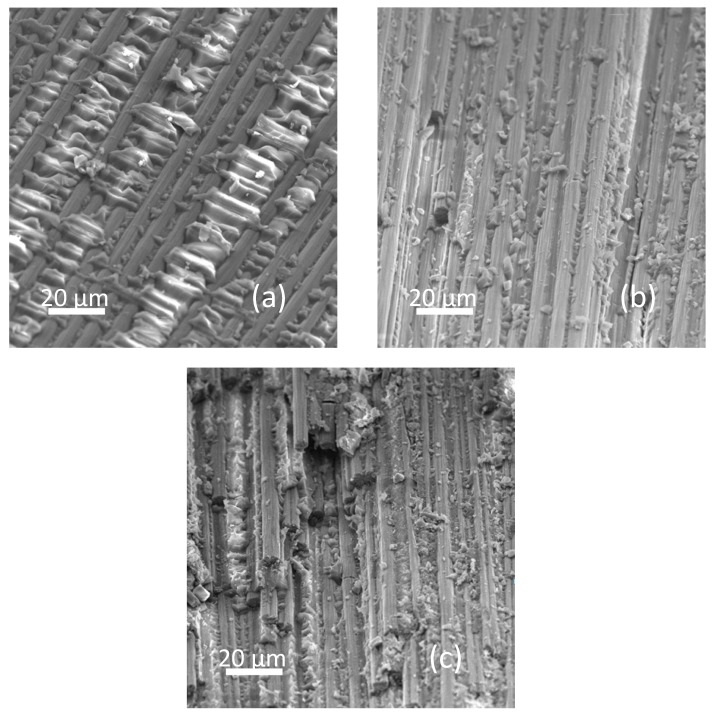
The scanning electron microscopic images of the fracture surfaces of laminate samples with (**a**) 0 wt%, (**b**) 1 wt% and (**c**) 5 wt%.

To further investigate the influence of GrF content on the composite matrix, the scanning electron microscopic images of the fracture surfaces of laminate samples are shown in Figure 8. The pure sample exhibits an intact and smooth fracture surface, because the fracture incidence is almost fully caused by the brittleness of the matrix. For the 1 wt% sample, there no smooth areas can be observed—instead the fracture surface becomes granular, and the fracture incidence starts to trigger the crack propagation due to the incorporated filler generated microvoids. When the microvoids are at a low concentration, the distance between each one is not adequate to form a coalescence to produce the primary crack—meanwhile the crack pinning and crack deflection can increase the fracture toughness, thus the composites sample at 1 wt% presents the highest impact resistance. However, at 5 wt% the composites sample has much higher concentration of microvoids whose shorter mutual distance facilitates the formation of microcrack coalescence and then promote the major crack propagation, the crack pinning and crack deflection still make reinforcement but the primary crack propagation effect dominates the composites, so the fracture toughness is lowered, and the fracture surface of 5 wt% becomes more granular.

### 3.3. Residual Strength upon CAI Tests

**Figure 9:**
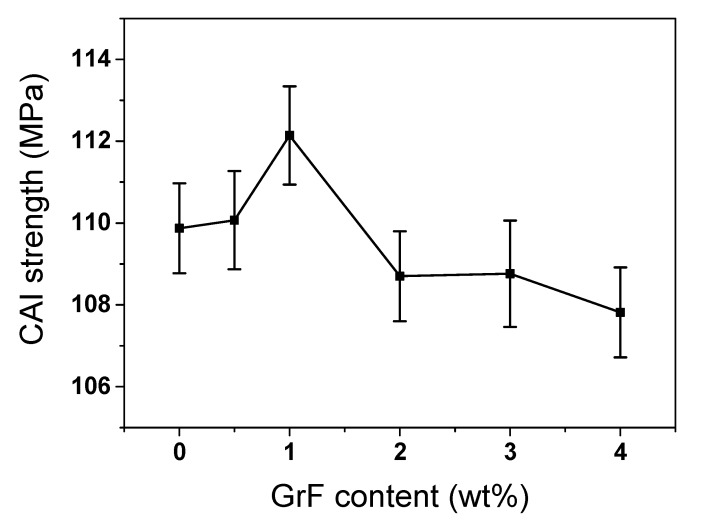
The CAI strength of load and average maximum load vs. GrF content curves of CFRP composites.

The residual strength after impact was characterized using the CAI test on the basis of the ASTM D7137M standard. Under the same conditions of laminate lay-ups (namely, the carbon fiber and epoxy matrix types, the ply number and laminate thickness), the value of CAI strength relies upon the method of lay-ups to a great extent, such as plain-woven, cross-ply or quasi-isotropic, etc. Sanchez-Saez et al. found that when the orientation of carbon fiber lay-ups was more isotropic, the compression residual strength was larger [42]—under the normalized strength condition, the order of compression residual strength reduction level was cross-ply > woven > quasi-isotropic. Although the woven pattern is efficiently resistant to the delamination extension compared to cross-ply laminates, the quasi-isotropic laminates have the largest CAI damage tolerance among all lay-up types. In our case, all CFRP laminates adopt the same lay-ups conditions including fiber and epoxy matrix types, ply number, laminate thickness and lay-ups pattern, etc. The only difference is the incorporated GrF content variation. As previously mentioned, the GrF content variation gives rise to the matrix fracture toughness fluctuation, then causes different impact resistances of the CFRP composite laminates. The impact resistance differences produce different delamination areas which are responsible for the variation of compression residual strength reduction. During the compression load being carried out, the delamination propagates mainly perpendicularly to the loading direction [43], the larger delamination area, the more severe delamination propagation. In Figure 9, the compression residual strength is plotted versus the GrF content. Unsurprisingly, the 1 wt% sample has highest CAI strength, the CAI strength increases until 1 wt% and then deflects to decrease with the enhance of GrF content. The CAI strength variation is accordant with the absorbed energy, incipient damage load, maximum load and delamination variations. At a low GrF content, the CAI strength of CFRP composite laminates were reinforced; however, a higher GrF content could lower the CAI strength, so at 1 wt% the CAI strength curve forms a deflection point, and the 1 wt% sample become the sample which possesses the highest compression residual strength.

## 4. Conclusions

To evaluate the influence of GrF content on the low velocity impact resistance of the incorporated CFRP composite, a series of characterizations were carried out, including a drop-weight impact test, an ultrasonic C-scan evaluation and a compression after impact test, etc. According to the load vs. time curves of the characterized samples, the 1 wt% and 2 wt% samples had the highest contact stiffness among all the sample contents. The load-displacement curves imply that the absorbed energy varies with an increase in GrF content. The 1 wt% sample had the lowest absorbed energy, which corresponds to the lowest damage level. The absorbed energy vs. GrF content curve depicts a deflection at 1 wt% content, where at lower than 1 wt% the absorbed energy decreases, and at higher than 1 wt% the absorbed energy increases largely. The mechanism behind this behavior is the GrF content variation, caused crack pinning, crack deflection and crack propagation effects interaction. When the GrF content is low, the crack pinning and crack deflection effects predominate to toughen the epoxy matrix, whereas with the increase in GrF content the major crack propagation overwhelms the toughening effect, then the fracture toughness decreases. The fracture toughness is closely correlated with the impact resistance property. The curves of incipient damage load and maximum load also display a deflection point at 1 wt% content, which verifies this mechanism. The delamination area was measured using the C-scan technique. The tendency of the delamination area coincides with the absorbed energy variation, which presents a prospective quantitative dependence. The CAI test indicates that the 1 wt% sample has the highest compression residual strength, and contents lower or higher than 1 wt% displays a descending trend. In conclusion, during the competitive interaction of toughening effect and crack propagation effect, the 1 wt% GrF-modified CFRP has the most impact-resistant property and the highest impact tolerance.
